# Accurate categorisation of menopausal status for research studies: a step-by-step guide and detailed algorithm considering age, self-reported menopause and factors potentially masking the occurrence of menopause

**DOI:** 10.1186/s13104-022-05970-z

**Published:** 2022-03-04

**Authors:** Sarsha Yap, Amy Vassallo, David E. Goldsbury, Usha Salagame, Louiza Velentzis, Emily Banks, Dianne L. O’Connell, Karen Canfell, Julia Steinberg

**Affiliations:** 1 The Daffodil Centre, The University of Sydney, A Joint Venture with Cancer Council NSW, Sydney, Australia; 2 Cancer Council NSW, Sydney, NSW Australia; 3 The George Institute for Global Health, University of New South Wales, Sydney, NSW Australia; 4 Sydney School of Public Health, Faculty of Medicine and Health, University of Sydney, Sydney, Australia; 5 Centre for Health Record Linkage, Centre of Epidemiology and Evidence, NSW Health, Sydney, Australia; 6 Melbourne School of Population and Global Health, University of Melbourne, Victoria, 3010 Australia; 7 National Centre for Epidemiology and Population Health, Australian National University, Canberra, Australia; 8 School of Medicine and Public Health, University of Newcastle, Newcastle, Australia

**Keywords:** Menopause, Hysterectomy, Oophorectomy, Menopausal hormone therapy, 45 and Up Study, Menopausal status, Algorithm

## Abstract

**Objective:**

Menopausal status impacts risk for many health outcomes. However, factors including hysterectomy without oophorectomy and Menopausal Hormone Therapy (MHT) can mask menopause, affecting reliability of self-reported menopausal status in surveys. We describe a step-by-step algorithm for classifying menopausal status using: directly self-reported menopausal status; MHT use; hysterectomy; oophorectomy; intervention timing; and attained age. We illustrate this approach using the Australian 45 and Up Study cohort (142,973 women aged ≥ 45 years).

**Results:**

We derived a detailed seven-category menopausal status, able to be further consolidated into four categories (“pre-menopause”/“peri-menopause”/“post-menopause”/“unknown”) accounting for participants’ ages. 48.3% of women had potentially menopause-masking interventions. Overall, 93,107 (65.1%), 9076 (6.4%), 17,930 (12.5%) and 22,860 (16.0%) women had a directly self-reported “post-menopause”, “peri-menopause”, “pre-menopause” and “not sure”/missing status, respectively. 61,464 women with directly self-reported “post-menopause” status were assigned a “natural menopause” detailed derived status (menopause without MHT use/hysterectomy/oophorectomy). By accounting for participants’ ages, 105,817 (74.0%) women were assigned a “post-menopause” consolidated derived status, including 15,009 of 22,860 women with “not sure”/missing directly self-reported status. Conversely, 3178 of women with directly self-reported “post-menopause” status were assigned “unknown” consolidated derived status. This algorithm is likely to improve the accuracy and reliability of studies examining outcomes impacted by menopausal status.

**Supplementary Information:**

The online version contains supplementary material available at 10.1186/s13104-022-05970-z.

## Introduction

Menopause, the permanent cessation of ovulation and end of menstruation, is an important factor influencing risk of many conditions including gynaecological cancers and fracture [1–3]. Menopausal status is a key indicator of endogenous hormonal milieu, thus of interest in many health studies. Surveys often ask women to directly self-report whether they have experienced menopause. However, determining a woman’s true hormonal and menopausal status is complex, as certain interventions can affect menstrual bleeding and mask menopausal status: e.g. a hysterectomy without oophorectomy can cause the end of menstrual bleeding without stopping ovulation and monthly hormonal fluctuations, and menopausal hormone therapy (MHT) can cause bleeding after menopause [4, 5]. Inadequately accounting for underlying menopausal status can result in inaccurate findings from studies investigating exposures and outcomes related to menopause. For example, the effects of MHT on breast cancer risk can only be ascertained accurately in women who are postmenopausal, so are likely to be inaccurate if women with unknown/masked menopausal status are included, and underestimated if time since menopause is not accounted for appropriately [6, 7]. Additionally, excluding all women who have potentially masked/unknown menopause can reduce statistical power [8]. Some studies have developed algorithms to derive menopausal status accounting for MHT use, hysterectomy, or bilateral oophorectomy [6, 8–11], but as menopausal status is one of many risk factors analysed, often do not describe how the algorithm was developed and how specific coding decisions (e.g. age thresholds) affect the final assigned menopausal status. The STRAW+10 criteria enable an in-depth classification of reproductive stage, but rely on collection of detailed information on menstrual cycle variability (including length and flow), supplemented by repeated measurements of selected endocrine markers and antral follicle count for women with hysterectomy, which are generally unavailable in cohort studies [12]. In other research, a continuous reproductive ageing score was derived using fuzzy mathematics, which was then validated with interviews and sex hormone measurements, but a significant number of women taking exogenous hormones were excluded [13].

Here, we outline a detailed step-by-step approach to optimise data availability for analyses by more accurately deriving menopausal status, accounting for directly self-reported menopause, MHT use, hysterectomy, oophorectomy, their relative timing, and individuals’ age. We illustrate the application of this algorithm using data from the Australian 45 and Up Study. We compare self-reported menopausal status to (1) a detailed (seven-category) derived menopausal status, and (2) a consolidated (four-category) derived menopausal status. We report estimated prevalence of post-, peri-, and pre-menopausal women in the cohort based on these classifications.

### Data used to derive menopausal status

The Sax Institute’s 45 and Up Study cohort includes 267,153 New South Wales (NSW) residents aged ≥ 45 years recruited in 2006–2009 (142,973 women: median age 59.9 at recruitment, interquartile range [IQR]: 52.8–69.0). Participants were randomly sampled from the Services Australia (formerly the Australian Government Department of Human Services) Medicare enrolment database, which has near-complete coverage of the Australian population. About 18% of invitees participated, representing ~ 11% of the NSW population aged ≥ 45 years. Participants completed a baseline questionnaire providing socio-demographic, health and lifestyle information and consented to linkage of their records to population-wide health databases. Study details are described elsewhere [14].

## Main text

We considered the following self-reported characteristics from the 45 and Up Study baseline questionnaire: (1) directly self-reported menopausal status (“Have you been through menopause?”—“no”, “not sure (because hysterectomy, taking MHT, etc)”, “periods have become irregular”, “yes”); (2) MHT use (“never”, “former”, “current”); ever had a (3) hysterectomy (“yes”, “no”) or a (4) bilateral oophorectomy (“yes”, “no”); age at (5) baseline, and where applicable, at (6) menopause, (7) start of MHT use, (8) hysterectomy, (9) oophorectomy. Details of corresponding questionnaire items and age calculations are shown in Additional file 1. We excluded current hormonal contraceptive use in the algorithm as the number of current users at baseline was small (2903, 2% of all women); sensitivity analyses verified that excluding current hormonal contraceptive users did not change the final proportion of women who were classified as post-, peri-, or pre-menopausal (results not shown).

The algorithm for assigning menopausal status comprises multiple steps.

### Step 1: Determine intervention status based on MHT use, hysterectomy and bilateral oophorectomy

We mapped 13 combinations of interventions that could affect or mask menopausal status, assigning each woman to a corresponding category (Additional file 2). The proportions of women in these categories ranged from < 1% (oophorectomy, current MHT user) to 46.9% (no interventions).

### Step 2: Create a detailed derived menopausal status variable based on self-reported menopause, intervention status and timing

We constructed the detailed derived seven-category menopausal status variable by combining information from the category assignment in step 1 and directly self-reported menopausal status using the criteria shown in Additional file 3. Briefly, age at menopause, age started MHT and age at oophorectomy and/or hysterectomy were compared to determine the order of events. The intervention status and order of events were then used to assign women to one of the seven menopausal categories: “natural menopause”, “peri-menopause”, “pre-menopause”, “unknown”, “started MHT before period stopped”, “no periods due to hysterectomy”, “menopause from oophorectomy”. The age at menopause is unknown for women who responded “not sure” to menopause, so they were assigned to a category by prioritising any self-reported interventions in the following order: bilateral oophorectomy, current MHT use, and hysterectomy. Women with inconsistent responses or unknown age(s) for key interventions were assigned “unknown” status.

### Step 3: Consolidate derived menopausal status from seven to four categories: apply an age threshold to re-assign women highly likely to be post-menopausal

#### Part A: Determine an age threshold for natural menopause

As timing of menopause can differ between populations and/or time periods [3, 15], we determined at which age threshold women would be highly likely to be post-menopausal using the cohort data.

We considered three approaches for determining an age threshold, following previous studies [6, 8–10]. To ensure the determined age thresholds were unaffected by interventions, the threshold calculations only included women who had never used MHT, did not have bilateral oophorectomy nor hysterectomy, and were not using hormonal contraceptives at baseline.

For our reference method, we considered the age at menopause reported by women who were post-menopausal with a detailed derived status of “natural menopause”. Following a previous approach [10], we excluded women aged < 55 years at baseline as they could potentially misinterpret a temporary irregularity as menopause. Among women aged ≥ 55 years (median age 65.1 years), > 90% were post-menopausal at ≥ 55 years (median age at natural menopause: 50 years, IQR 48–53; Additional file 4), which was taken as the age threshold for further menopausal status re-classification (see below). In the conservative approach, we applied the 90% threshold to single-year age groups rather than cumulative age groups. For single-year age groups from age 57 years onwards, 90% of women were post-menopausal (Additional file 5), so this was chosen as the conservative age threshold. In the least-conservative approach, rather than considering single years of age, we identified the age threshold (54 years) above which cumulatively 90% of all women in the cohort were assigned “natural menopause” status (Additional file 6).

#### Part B: Consolidate menopausal status

We consolidated the detailed seven categories into four categories (“post-menopause”, “peri-menopause”, “pre-menopause”, “unknown”). The age threshold was used to re-classify “unknown” or possibly masked menopausal status derived in step 2 (masked status including “started MHT before period stopped”, “no periods due to hysterectomy”, “menopause from oophorectomy” without self-reported menopause as this combination potentially indicates unilateral oophorectomy). These women were assigned “post-menopause” status if their age at baseline was above the age threshold for the reference approach (or separately, the conservative or least-conservative approach), and “unknown” status otherwise. All women with “menopause from bilateral oophorectomy” status and self-reported menopause were assigned consolidated “post-menopause” status.

### Results

At least 48.3% of women in the 45 and Up Study reported at least one intervention that could affect menopause (Additional file 2).

#### Self-reported versus detailed derived menopausal status

**Fig. 1:**
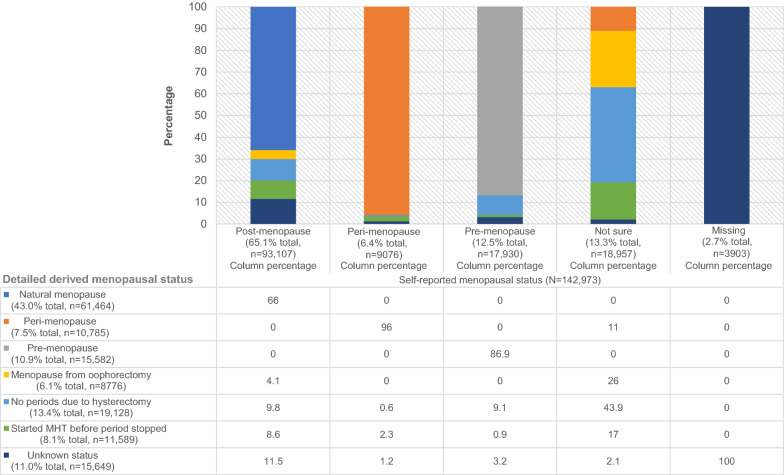
Self-reported menopausal status compared to the detailed derived menopausal status (taking into account interventions that could impact menopause) in the 45 and Up Study (n = 142,973)

93,107 women had directly self-reported “post-menopause” status (Fig. 1); of these, 61,464 (66.0%) and 3845 (4.1%) were assigned detailed derived “natural menopause” and “menopause from oophorectomy” status, respectively, while 17,125 (18.4%) were assigned a potentially masked detailed derived status. Of women with directly self-reported “peri-menopause”, “pre-menopause”, or “not sure” status, 262 (2.9%), 1784 (10.0%) and 11,546 (60.9%), respectively, were assigned a potentially masked detailed derived status. Furthermore, 10,673 (11.5%), 105 (1.2%) and 564 (3.2%) women with directly self-reported “post-menopause”, “peri-menopause” or “pre-menopause” status, respectively, were assigned a detailed derived “unknown” status due to inconsistent responses or unknown age(s) for key interventions.

#### Self-reported versus consolidated derived menopausal status

**Fig. 2:**
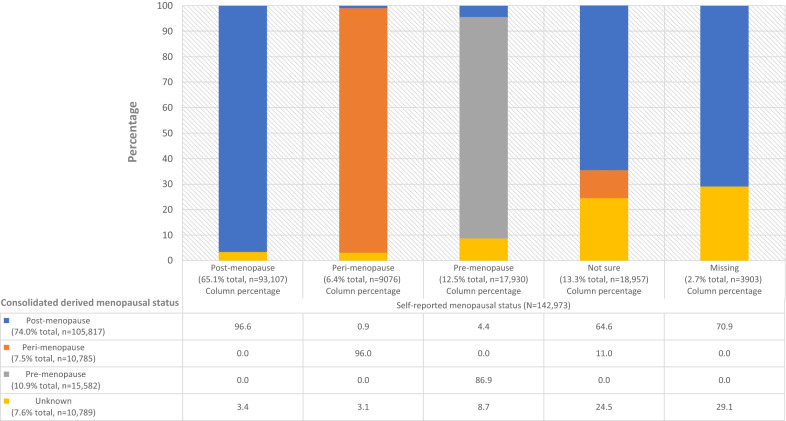
Self-reported menopausal status compared to consolidated derived menopausal status (taking into account interventions that could impact menopause and age at baseline using the reference approach)

Overall, 93,107 of women had directly self-reported “post-menopause” status compared to 105,817 of women with consolidated derived “post-menopause” status based on the reference approach (+ 13.7% relative increase at the reference approach threshold of age ≥ 55 years at baseline; Table 1). A small proportion (3.4%, 3,178) of women with directly self-reported “post-menopause” status were assigned “unknown” consolidated derived status due to interventions or missing information on their timing (Fig. 2). By contrast, 15,888 of women with directly self-reported status other than “post-menopause” were assigned “post-menopause” consolidated derived status. Finally, only 10,789 women had “unknown” consolidated derived status, compared to 22,860 women with “not sure”/missing directly self-reported status (− 52.8%). Frequencies for “peri-menopause” or “pre-menopause” status between the self-reported and derived status were very similar. Additional file 7 shows the correspondence between directly self-reported, detailed derived and consolidated derived menopausal status.

**Table 1:** Self-reported and consolidated derived menopausal status for women in the 45 and Up Study

Menopausal status	Post-menopause	Peri-menopause	Pre-menopause	Unknown
	n (% total; % change compared to self-report)	n (% total; % change compared to self-report)	n (% total; % change compared to self-report)	n (% total; % change compared to self-report)
Self-reported	93,107 (65.1)	9,076 (6.4)	17,930 (12.5)	22,860 (16.0)
Consolidated—reference method^a^	105,817 (74.0%; + 8.9%)	10,785 (7.5%; + 1.2%)	15,582 (10.9%; − 1.6%)	10,789 (7.6%; − 8.4%)
Consolidated—conservative method^b^	102,008 (71.4%; + 6.2%)	10,785 (7.5%; + 1.2%)	15,582 (10.9%; − 1.6%)	14,598 (10.2%; − 5.8%)
Consolidated—least-conservative method^c^	107,394 (75.1%; + 10.0%)	10,785 (7.5%; + 1.2%)	15,582 (10.9%; − 1.6%)	9,212 (6.4%; − 9.6%)
Consolidated—reference method without re-classifying women with detailed “unknown” status	92,629 (62.7%; − 0.3%)	10,785 (7.5%; + 1.2%)	15,582 (10.9%; − 1.6%)	23,977 (16.8%; + 0.8%)
Consolidated—conservative method without re-classifying women with detailed “unknown” status	89,683 (65.6%; − 2.4%)	10,785 (7.5%; + 1.2%)	15,582 (10.9%; − 1.6%)	26,923 (18.8%; + 2.8%)
Consolidated—least-conservative method without re-classifying women with detailed “unknown” status	93,849 (65.6%; + 0.5%)	10,785 (7.5%; + 1.2%)	15,582 (10.9%; − 1.6%)	22,757 (15.9%; − 0.1%)

^a^Unknown or possibly masked status re-classified to “post-menopause” status for women aged ≥ 55 years at baseline

^b^Unknown or possibly masked status re-classified to “post-menopause” status for women aged ≥ 57 years at baseline

^c^Unknown or possibly masked status re-classified to “post-menopause” status for women aged ≥ 54 years at baseline

The three different age-thresholds to obtain the consolidated derived status produced similar results for all menopausal status categories (< 5% difference based on age thresholds ≥ 54, ≥ 55 and ≥ 57 years at baseline; Table 1). For all methods, the largest changes compared to self-reported menopausal status were due to re-classifying women with consolidated derived “unknown” status; exclusion of these women from re-classification gave results similar to self-reported menopausal status (< 3% difference).

### Discussion

We developed a detailed algorithm for assignment of likely underlying menopausal status. Of women with self-reported menopause, only 66.0% were assigned “natural menopause” detailed derived status, while 29.9% were assigned potentially masked or “unknown” detailed derived status. Consolidation of the detailed derived menopausal status into four categories using the reference approach (here, age ≥ 55 years at baseline) increased the total number of women who were classified as post-menopausal by 13.7% compared to directly self-reported status, while re-classifying 3.4% of women with self-reported “post-menopause” status as “unknown”. The consolidated derived status also included more than halved the number of women with unknown menopausal status compared to directly self-reported “not sure”/missing status.

Improved classification of menopausal status can yield more reliable results and increase statistical power for health studies. Notably, many studies (e.g. for health impacts of MHT) need to restrict analyses to post-menopausal women, so benefit from an algorithm allowing both evidence-based classification of natural menopause and reliable identification of a larger number of women highly likely to be post-menopausal.

The 45 and Up Study covers age groups for which menopausal status is of key interest. Median self-reported age at menopause was 50 years (based on women aged ≥ 55 years at baseline who had no hysterectomy, no bilateral oophorectomy, never used MHT and were not using hormonal contraceptives at baseline), comparable to the median age at natural menopause of 50–51 years reported in three other, smaller Australian studies (~ 500–25,000 participants) [15]. The prevalence of hysterectomy, bilateral oophorectomy, and MHT use at recruitment are also comparable to estimates from other Australian studies (Additional file 8) [16, 17].

In the 45 and Up Study, all three approaches for determining age thresholds for natural menopause produced similar results, thus we recommend the reference approach (here, resulting in an age threshold of ≥ 55 years at baseline) as used by the Collaborative Group on Hormonal Factors in Breast Cancer [10]. For other surveys including women aged < 45 years, the age threshold approaches can be adapted to classify women as likely pre-menopausal. Depending on the study aims, cut-points higher or lower than 90% can also be used to determine age thresholds to e.g. increase the number of women classified as post-menopausal (but potentially decrease the specificity of the classification). The approach described here may be useful for other studies investigating women’s health.

## Limitations

MHT use, hysterectomy, and oophorectomy were based on self-report rather than linked medical records. However, self-reported data on many of these variables, including MHT use, have been shown to be highly accurate [18]. Information on endometrial ablation was not collected. The algorithm could not be validated against directly measured hormone levels. We also had to make assumptions to reconcile inconsistent responses to questions on menopause and the relevant interventions. For example, women who indicated they had a bilateral oophorectomy and responded “no” to self-reported menopause were assumed to have had a unilateral oophorectomy and were assigned a detailed derived status of “pre-menopause” (Additional file 3). This could lead to some misclassification. However, as the algorithm described here explicitly incorporates information on interventions that can mask or affect menopause, it can provide a more granular menopausal status assignment than self-report.

## Supplementary Information

**Additional file 1: **45 and Up Study baseline questionnaire data used in the algorithm for menopausal status assignment.

**Additional file 2: **Systematic categorisation of the combinations of interventions that could affect menopausal status and frequency of each category in the 45 and Up Study (n = 142,973). Final categories are shown in dark red.

**Additional file 3: **Assignment of the detailed derived menopausal status based on self-reported menopausal status, oophorectomy, hysterectomy, MHT use, and the timing of these interventions for women in the 45 and Up Study. Fewer than 5 women, actual number suppressed to preserve confidentiality.^ Missing either one or more responses to ever had a hysterectomy, oophorectomy, ever used MHT or self-reported menopause.

**Additional file 4: **Cumulative percentage of female 45 and Up Study participants with self-reported age at menopause (years) below each age threshold. This figure illustrates the approach used to determine the age threshold for the reference approach (here, age ≥ 55 years at baseline). The cumulative percentage of female participants with self-reported age at menopause below each age threshold is based on women aged 55 + years at baseline who had experienced natural menopause and reported their age at menopause, never used MHT, have not had an oophorectomy nor a hysterectomy, and were not using oral contraceptives at baseline (n = 30,591).

**Additional file 5: **Frequency of self-reported menopausal status of female 45 and Up Study participants by single year age at baseline. This table illustrates the approach used to determine the age threshold for the conservative approach (here, ≥ age 57 years at baseline). The frequency is based on female participants who had never used MHT, have not had an oophorectomy nor a hysterectomy, and did not use oral contraceptives at baseline (n = 63,742).

**Additional file 6: **Cumulative percentage of female 45 and Up Study participants who had experienced natural menopause by age at baseline. This figure illustrates the approach used to determine the age threshold for the least-conservative approach (here, age ≥ 54 years at baseline). The percentage is based on all female 45 and Up Study participants who had never used MHT, have not had an oophorectomy nor a hysterectomy, and were not using oral contraceptives at baseline (n = 41,891).

**Additional file 7: **Correspondence between directly self-reported, detailed derived and consolidated derived menopausal status in the 45 and Up Study (based on the reference approach, here, age ≥ 55 years at baseline). The diagram illustrates the classification of female 45 and Up Study participants by directly self-reported menopausal status (left vertical bar), detailed derived menopausal status (middle vertical bar) and consolidated derived menopausal status (right vertical bar). The horizontal bands represent the re-classification of participants between the different status assignments, coloured according to the directly self-reported menopausal status. For example, ~ 19,000 participants had directly self-reported “not sure” status (light blue horizontal bands), of whom ~ 8,000 were assigned the “no periods due to hysterectomy” detailed derived menopausal status. Of these ~ 5,500 were then assigned “post-menopause” consolidated derived status in the final step, while ~ 2,500 were assigned “unknown” consolidated derived status.

**Additional file 8: **Age at menopause, prevalence of hysterectomy, bilateral oophorectomy and current MHT use in the 45 and Up Study compared to other Australian studies. 1 Estimates from InterLACE Study Team. Variations in reproductive events across life: a pooled analysis of data from 505 147 women across 10 countries. Hum Reprod. 2019;34(5):881–93. 2 Estimates from Wilson LF, Pandeya N, Byles J, Mishra GD. Hysterectomy status and all-cause mortality in a 21-year Australian population-based cohort study. Am J Obstet Gynecol. 2019;220(1):83 e1–e11. 3 Estimates from Velentzis LS, Banks E, Sitas F, Salagame U, Tan EH, Canfell K. Use of Menopausal Hormone Therapy and Bioidentical Hormone Therapy in Australian Women 50 to 69 Years of Age: Results from a National, Cross-Sectional Study. PloS one. 2016;11(3):e0146494-e.

## Data Availability

Data used for this study were accessed using the Secure Unified Research Environment (SURE). Data from the 45 and Up Study are available from the Sax Institute but restrictions apply to their availability. Therefore the authors cannot on-provide the data to other researchers. Researchers are able to access these data from the relevant data custodians for approved research projects, and enquiries for data access can be made to the Sax Institute (see https://www.saxinstitute.org.au/our-work/45-up-study/for-researchers/ for details).

## Abbreviations

MHT: Menopausal hormone therapy
NSW: New South Wales
NSWCR: New South Wales Cancer Registry
