# Supplementary material for: Accurate categorisation of menopausal status for research studies: a step-by-step guide and detailed algorithm considering age, self-reported menopause and factors potentially masking the occurrence of menopause
Source: BMC Res Notes. 2022 Mar 4;15:88. doi: 10.1186/s13104-022-05970-z (PMC8895593; doi:10.1186/s13104-022-05970-z)
Supplement: Supplementary file 1 — Additional file 1: 45 and Up Study baseline questionnaire data used in the algorithm for menopausal status assignment. [file 13104_2022_5970_MOESM1_ESM.docx]

**Additional file 1: 45 and Up Study baseline questionnaire data used in the algorithm for menopausal status assignment.**

| **Characteristic** | **45 and Up baseline questionnaire item(s)** | **Algorithm step(s)** | **Use of characteristic in algorithm** |
| --- | --- | --- | --- |
| Hysterectomy | Have you ever had any of the following operations?  ─Hysterectomy | Step 1 | One of the characteristics used for deriving intervention status. See Figure 1. |
| ─no | ─responded no |  |  |
| ─yes | ─responded yes |  |  |
| Bilateral oophorectomy | Have you ever had any of the following operations?  ─Both ovaries removed | Step 1 | One of the characteristics used for deriving intervention status. See Figure 1. |
| ─no | ─responded no |  |  |
| ─yes | ─responded yes |  |  |
| Menopausal Hormone Therapy (MHT) use | Have you ever used hormone replacement therapy (HRT)? (yes or no) and Are you currently taking HRT? (yes or no) | Step 1 | One of the characteristics used for deriving intervention status. See Figure 1. |
| ─never | ─responded no |  |  |
| ─past MHT user | ─responded yes and no, respectively |  |  |
| ─current MHT user | ─responded yes and yes, respectively |  |  |
| ─missing |  |  |  |
| Self-reported menopause | Have you been through menopause? | Step 1 and Step 2 | Step 1: One of the characteristics used for deriving intervention status. See Figure 1. |
| ─post-menopause | ─yes |  | Step 2: One of the characteristics used for deriving detailed menopausal status. See Additional file 2. |
| ─peri-menopause | ─my periods have become irregular |  |  |
| ─pre-menopause | ─no |  |  |
| ─not sure | ─not sure (because hysterectomy, taking HRT, etc) |  |  |
| ─missing |  |  |  |
| Age had a hysterectomy | Have you ever had any of the following operations?  ─ Hysterectomy, age when had operation | Step 2 | Determining the order of events (i.e. menopause, started MHT, oophorectomy and/or hysterectomy), where applicable. See Additional file 2. |
| Age had a bilateral oophorectomy | Have you ever had any of the following operations?  ─ Both ovaries removed, age when had operation | Step 2 | Determining the order of events (i.e. menopause, started MHT, oophorectomy and/or hysterectomy), where applicable. See Additional file 2. |
| Age started menopausal hormone therapy (MHT) | Have you ever used hormone replacement therapy (HRT)?  ─If yes, for how long altogether have you used HRT, and Are you currently taking HRT? ─If no, at what age did you stop | Step 2 | Determining the order of events (i.e. menopause, started MHT, oophorectomy and/or hysterectomy), where applicable. See Additional file 2. |
| ─past MHT user | ─age started MHT calculated as age stopped MHT minus number of years MHT was used |  |  |
| ─current MHT user | ─age started MHT calculated as age at baseline minus number of years MHT was used |  |  |
| Age at menopause | Have you been through menopause? ─If yes, how old were you when you went through menopause | Step 2 and Step 3 | Step 2: Determining the order of events (i.e. menopause, started MHT, oophorectomy and/or hysterectomy), where applicable. See Additional file 2. |
|  |  | Step 3 | Step 3: Determining the age threshold for natural menopause using the reference method. See Additional file 3. |
| Attained age | What is your date of birth? and What is today's date? | Step 3 | Step 3A: Determining the age threshold for natural menopause using the conservative method. See Additional file 4. |
| (age at baseline) | ─attained age calculated as (today's date - date of birth)/365.25 |  | Step 3A: Determining the age threshold for natural menopause using the least conservative method. See Additional file 5. |
|  |  |  | Step 3B: Re-classifying women with unknown or possibly masked detailed derived status as post-menopausal, if their attained age was equal or above the age threshold for the different methods |
