# Supplementary material for: Accurate categorisation of menopausal status for research studies: a step-by-step guide and detailed algorithm considering age, self-reported menopause and factors potentially masking the occurrence of menopause
Source: BMC Res Notes. 2022 Mar 4;15:88. doi: 10.1186/s13104-022-05970-z (PMC8895593; doi:10.1186/s13104-022-05970-z)
Supplement: Supplementary file 2 — Additional file 2: Systematic categorisation of the combinations of interventions that could affect menopausal status and frequency of each category in the 45 and Up Study (n = 142,973). Final categories are shown in dark red. [file 13104_2022_5970_MOESM2_ESM.docx]

**45 and Up Study female participants (n=142,973)**

**Missing a response for:**

**self-reported menopause, MHT use, ever had hysterectomy or oophorectomy.**

**Yes (n=6,818, 4.8%)**

**No (n=136,155)**

**13. Unknown**

**Hysterectomy**

**No (n=97,521)**

**Yes (n=38,634)**

**Oophorectomy**

**Oophorectomy**

**No (n=96,080)**

**Yes (n=1,441)**

**No (n=26,647)**

**Yes (n=11,987)**

**Ever used MHT.**

**Ever used MHT.**

**Ever used MHT.**

**Ever used MHT.**

**No (n=67,035, 46.9%)**

**Yes (n=29,045)**

**No (n=807, 0.6%)**

**Yes (n=634)**

**No (n=13,023, 9.1%)**

**Yes (n=13,624)**

**No (n=3,529, 2.5%)**

**Yes (n=8,458)**

**Currently using MHT**

**Currently using MHT**

**Currently using MHT**

**Currently using MHT**

**1. No interventions**

**4. Oophorectomy**

**7. Hysterectomy**

**10. Hysterectomy and Oophorectomy**

**No/Missing (n=22,284, 15.6%)**

**Yes (n=6,761, 4.7%)**

**No/Missing (n=471, 0.3%)**

**Yes (n=163, 0.1%)**

**No/Missing (n=9,497, 6.6%)**

**Yes (n=4,127, 2.9%)**

**No/Missing (n=5,588, 3.9%)**

**Yes (n=2,870, 2.0%)**

**2. Past MHT user**

**3. Current MHT user**

**5. Oophorectomy, Past MHT user**

**6. Oophorectomy, Current MHT user**

**9. Hysterectomy, Current MHT user**

**8. Hysterectomy, Past MHT user**

**11. Hysterectomy, Oophorectomy and past MHT user**

**12. Hysterectomy, Oophorectomy and current MHT user**

**Additional file 2: Systematic categorisation of the combinations of interventions that could affect menopausal status and frequency of each category in the 45 and Up Study (n=142,973). Final categories are shown in dark red.**
