# Supplementary material for: Accurate categorisation of menopausal status for research studies: a step-by-step guide and detailed algorithm considering age, self-reported menopause and factors potentially masking the occurrence of menopause
Source: BMC Res Notes. 2022 Mar 4;15:88. doi: 10.1186/s13104-022-05970-z (PMC8895593; doi:10.1186/s13104-022-05970-z)
Supplement: Supplementary file 3 — Additional file 3: Assignment of the detailed derived menopausal status based on self-reported menopausal status, oophorectomy, hysterectomy, MHT use, and the timing of these interventions for women in the 45 and Up Study. Fewer than 5 women, actual number suppressed to preserve confidentiality.^ Missing either one or more responses to ever had a hysterectomy, oophorectomy, ever used MHT or self-reported menopause. [file 13104_2022_5970_MOESM3_ESM.docx]

**Additional file 3. Assignment of the detailed derived menopausal status based on self-reported menopausal status, oophorectomy, hysterectomy, MHT use, and the timing of these interventions for women in the 45 and Up Study.**

| **Criteria** | | | | | | **Number of women (N=142,973)** | **Assigned detailed derived menopausal status** |
| --- | --- | --- | --- | --- | --- | --- | --- |
| **Intervention status (see** **Figure 1)** | **Hysterectomy** | **Bilateral  oophorectomy** | **MHT user** | **Self-reported menopausal status: “**Have you been through menopause?” | **Timing of interventions** |  |  |
| 1. No interventions (n=67,035, 46.9%) | No | No | Never | Yes (“post-menopause”) | None | 42090 | Natural menopause |
|  |  |  |  | Irregular (“peri-menopause”) | None | 8299 | Peri-menopause |
|  |  |  |  | No (“pre-menopause”) | None | 1481 | Pre-menopause |
|  |  |  |  | Not sure (“part of unknown”) | None | 15165 | Peri-menopause |
| 2. Past MHT user (n=22,284, 15.6%) | No | No | Past | Yes (“post-menopause”) | Age at menopause or started MHT missing | 3284 | Unknown |
|  |  |  |  |  | Age menopause ≤ age started MHT | 11416 | Natural menopause |
|  |  |  |  |  | Age menopause > age started MHT | 6298 | Started MHT before period stopped |
|  |  |  |  | Irregular (“peri-menopause”) | None | 366 | Peri-menopause |
|  |  |  |  | No ("pre-menopause") | None | 325 | Pre-menopause |
|  |  |  |  | Not sure (“part of unknown”) | None | 595 | Peri-menopause |
| 3. Current MHT user (n=6,761, 4.7%) | No | No | Current | Yes (“post-menopause”) | Age at menopause or started MHT missing | 395 | Unknown |
|  |  |  |  |  | Age menopause ≤ age started MHT | 3654 | Natural menopause |
|  |  |  |  |  | Age menopause > age started MHT | 1044 | Started MHT before period stopped |
|  |  |  |  | Irregular (“peri-menopause”) | None | 207 | Started MHT before period stopped |
|  |  |  |  | No ("pre-menopause") | None | 151 | Started MHT before period stopped |
|  |  |  |  | Not sure (“part of unknown”) | None | 1310 | Started MHT before period stopped |
| 4. Bilateral oophorectomy (n=807, 0.6%) | No | Yes | Never | Yes (“post-menopause”) | Age at menopause or oophorectomy missing | 84 | Unknown |
|  |  |  |  |  | Age menopause < age oophorectomy | 183 | Natural menopause |
|  |  |  |  |  | Age menopause ≥ age oophorectomy | 361 | Menopause from oophorectomy |
|  |  |  |  | Irregular (“peri-menopause”) | None | 41 | Peri-menopause |
|  |  |  |  | No ("pre-menopause") | None | 82 | Pre-menopause |
|  |  |  |  | Not sure (“part of unknown”) | None | 56 | Menopause from oophorectomy |
| 5. Bilateral oophorectomy, Past MHT user (n=471, 0.3%) | No | Yes | Past | Yes (“post-menopause”) | Age at menopause or oophorectomy or started MHT missing | 117 | Unknown |
|  |  |  |  |  | Age menopause < age oophorectomy, | 75 | Natural menopause |
|  |  |  |  |  | Age menopause ≤ age started MHT |  |  |
|  |  |  |  |  | Age menopause < age oophorectomy, | 29 | Started MHT before period stopped |
|  |  |  |  |  | Age menopause > age started MHT |  |  |
|  |  |  |  |  | Age menopause ≥ age oophorectomy | 198 | Menopause from oophorectomy |
|  |  |  |  | Irregular (“peri-menopause”) | None | * | Peri-menopause |
|  |  |  |  | No ("pre-menopause") | None | * | Pre-menopause |
|  |  |  |  | Not sure (“part of unknown”) | None | 39 | Menopause from oophorectomy |
| 6. Bilateral oophorectomy, Current MHT user (n=163, 0.1%) | No | Yes | Current | Yes (“post-menopause”) | Age at menopause or oophorectomy or started MHT missing | 16 | Unknown |
|  |  |  |  |  | Age menopause < age oophorectomy, | 23 | Natural menopause |
|  |  |  |  |  | Age menopause ≤ age started MHT |  |  |
|  |  |  |  |  | Age menopause < age oophorectomy, | 8 | Started MHT before period stopped |
|  |  |  |  |  | Age menopause > age started MHT |  |  |
|  |  |  |  |  | Age menopause ≥ age oophorectomy | 69 | Menopause from oophorectomy |
|  |  |  |  | Irregular (“peri-menopause”) | None | * | Started MHT before period stopped |
|  |  |  |  | No ("pre-menopause") | None | * | Started MHT before period stopped |
|  |  |  |  | Not sure (“part of unknown”) | None | 42 | Menopause from oophorectomy |
| 7. Hysterectomy (n=13,023, 9.1%) | Yes | No | Never | Yes (“post-menopause”) | Age at menopause or hysterectomy missing | 1063 | Unknown |
|  |  |  |  |  | Age menopause < age hysterectomy | 1574 | Natural menopause |
|  |  |  |  |  | Age menopause ≥ age hysterectomy | 3620 | No periods due to hysterectomy |
|  |  |  |  | Irregular (“peri-menopause”) | None | 31 | No periods due to hysterectomy |
|  |  |  |  | No ("pre-menopause") | None | 1441 | No periods due to hysterectomy |
|  |  |  |  | Not sure (“part of unknown”) | None | 5294 | No periods due to hysterectomy |
| 8. Hysterectomy, Past MHT user (n=9,497, 6.6%) | Yes | No | Past | Yes (“post-menopause”) | Age at menopause, or hysterectomy or started MHT missing | 1417 | Unknown |
|  |  |  |  |  | Age menopause < age hysterectomy, | 828 | Natural menopause |
|  |  |  |  |  | Age menopause ≤ age started MHT |  |  |
|  |  |  |  |  | Age menopause < age hysterectomy, | 319 | Started MHT before periods stopped |
|  |  |  |  |  | Age menopause > age started MHT |  |  |
|  |  |  |  |  | Age menopause ≥ age hysterectomy | 3778 | No periods due to hysterectomy |
|  |  |  |  | Irregular (“peri-menopause”) | None | 5 | No periods due to hysterectomy |
|  |  |  |  | No ("pre-menopause") | None | 126 | No periods due to hysterectomy |
|  |  |  |  | Not sure (“part of unknown”) | None | 3024 | No periods due to hysterectomy |
| 9. Hysterectomy, Current MHT user (n=4,127, 2.9%) | Yes | No | Current | Yes (“post-menopause”) | Age at menopause, or hysterectomy or started MHT missing | 328 | Unknown |
|  |  |  |  |  | Age menopause < age hysterectomy, | 315 | Natural menopause |
|  |  |  |  |  | Age menopause ≤ age started MHT |  |  |
|  |  |  |  |  | Age menopause < age hysterectomy, | * | Started MHT before periods stopped |
|  |  |  |  |  | Age menopause > age started MHT |  |  |
|  |  |  |  |  | Age menopause ≥ age hysterectomy | 1449 | No periods due to hysterectomy |
|  |  |  |  | Irregular (“peri-menopause”) | None | * | No periods due to hysterectomy |
|  |  |  |  | No ("pre-menopause") | None | 62 | No periods due to hysterectomy |
|  |  |  |  | Not sure (“part of unknown”) | None | 1918 | Started MHT before period stopped |
| 10. Hysterectomy, Bilateral oophorectomy (n=3,529, 2.5%) | Yes | Yes | Never | Yes (“post-menopause”) | Age at menopause or hysterectomy or oophorectomy missing | 557 | Unknown |
|  |  |  |  |  | Age menopause < age oophorectomy, | 600 | Natural menopause |
|  |  |  |  |  | Age menopause < age hysterectomy |  |  |
|  |  |  |  |  | Age menopause < age oophorectomy, | 70 | No periods due to hysterectomy |
|  |  |  |  |  | Age menopause ≥ age hysterectomy |  |  |
|  |  |  |  |  | Age menopause ≥ age oophorectomy | 948 | Menopause from oophorectomy |
|  |  |  |  | Irregular (“peri-menopause”) | None | 9 | No periods due to hysterectomy |
|  |  |  |  | No ("pre-menopause") | None | 181 | Unknown |
|  |  |  |  | Not sure (“part of unknown”) | None | 1164 | Menopause from oophorectomy |
| 11. Hysterectomy, Bilateral oophorectomy, Past MHT user (n=5,588, 3.9%) | Yes | Yes | Past | Yes (“post-menopause”) | Age at menopause or hysterectomy or oophorectomy or started MHT missing | 983 | Unknown |
|  |  |  |  |  | Age menopause < age oophorectomy, | 473 | Natural menopause |
|  |  |  |  |  | Age menopause < age hysterectomy, |  |  |
|  |  |  |  |  | Age menopause ≤ age started MHT |  |  |
|  |  |  |  |  | Age menopause < age oophorectomy, | 190 | Started MHT before period stopped |
|  |  |  |  |  | Age menopause < age hysterectomy, |  |  |
|  |  |  |  |  | Age menopause > age started MHT |  |  |
|  |  |  |  |  | Age menopause < age oophorectomy, | 150 | No periods due to hysterectomy |
|  |  |  |  |  | Age menopause ≥ age hysterectomy |  |  |
|  |  |  |  |  | Age menopause ≥ age oophorectomy | 1629 | Menopause from oophorectomy |
|  |  |  |  | Irregular (“peri-menopause”) | None | 6 | No periods due to hysterectomy |
|  |  |  |  | No ("pre-menopause") | None | 79 | Unknown |
|  |  |  |  | Not sure (“part of unknown”) | None | 2078 | Menopause from oophorectomy |
| 12. Hysterectomy, Bilateral oophorectomy, Current MHT user (n=2,870, 2.0%) | Yes | Yes | Current | Yes (“post-menopause”) | Age at menopause or hysterectomy or oophorectomy or started MHT missing | 251 | Unknown |
|  |  |  |  |  | Age menopause < age oophorectomy, | 233 | Natural menopause |
|  |  |  |  |  | Age menopause < age hysterectomy, |  |  |
|  |  |  |  |  | Age menopause ≤ age started MHT |  |  |
|  |  |  |  |  | Age menopause < age oophorectomy, | 58 | Started MHT before period stopped |
|  |  |  |  |  | Age menopause < age hysterectomy, |  |  |
|  |  |  |  |  | Age menopause > age started MHT |  |  |
|  |  |  |  |  | Age menopause < age oophorectomy, | 60 | No periods due to hysterectomy |
|  |  |  |  |  | Age menopause ≥ age hysterectomy |  |  |
|  |  |  |  |  | Age menopause ≥ age oophorectomy | 640 | Menopause from oophorectomy |
|  |  |  |  | Irregular (“peri-menopause”) | None | 6 | Unknown |
|  |  |  |  | No ("pre-menopause") | None | 70 | Unknown |
|  |  |  |  | Not sure (“part of unknown”) | None | 1552 | Menopause from oophorectomy |
| 13. Unknown (n=6,818, 4.8%) | Missing^ | Missing^ | Missing^ | Missing^ | None | 6818 | Unknown |

Fewer than 5 women, actual number suppressed to preserve confidentiality.

^ Missing either one or more responses to ever had a hysterectomy, oophorectomy, ever used MHT or self-reported menopause.
