# Supplementary material for: Accurate categorisation of menopausal status for research studies: a step-by-step guide and detailed algorithm considering age, self-reported menopause and factors potentially masking the occurrence of menopause
Source: BMC Res Notes. 2022 Mar 4;15:88. doi: 10.1186/s13104-022-05970-z (PMC8895593; doi:10.1186/s13104-022-05970-z)
Supplement: Supplementary file 4 — Additional file 4: Cumulative percentage of female 45 and Up Study participants with self-reported age at menopause (years) below each age threshold. This figure illustrates the approach used to determine the age threshold for the reference approach (here, age ≥ 55 years at baseline). The cumulative percentage of female participants with self-reported age at menopause below each age threshold is based on women aged 55 + years at baseline who had experienced natural menopause and reported their age at menopause, never used MHT, have not had an oophorectomy nor a hysterectomy, and were not using oral contraceptives at baseline (n = 30,591). [file 13104_2022_5970_MOESM4_ESM.docx]

**Additional file 4. Cumulative percentage of female 45 and Up Study participants with self-reported age at menopause (years) below each age threshold.**

This figure illustrates the approach used to determine the age threshold for the reference approach (here, age ≥55 years at baseline). The cumulative percentage of female participants with self-reported age at menopause below each age threshold is based on women aged 55+ years at baseline who had experienced natural menopause and reported their age at menopause, never used MHT, have not had an oophorectomy nor a hysterectomy, and were not using oral contraceptives at baseline (n=30,591).
