# Supplementary material for: Accurate categorisation of menopausal status for research studies: a step-by-step guide and detailed algorithm considering age, self-reported menopause and factors potentially masking the occurrence of menopause
Source: BMC Res Notes. 2022 Mar 4;15:88. doi: 10.1186/s13104-022-05970-z (PMC8895593; doi:10.1186/s13104-022-05970-z)
Supplement: Supplementary file 5 — Additional file 5: Frequency of self-reported menopausal status of female 45 and Up Study participants by single year age at baseline. This table illustrates the approach used to determine the age threshold for the conservative approach (here, ≥ age 57 years at baseline). The frequency is based on female participants who had never used MHT, have not had an oophorectomy nor a hysterectomy, and did not use oral contraceptives at baseline (n = 63,742). [file 13104_2022_5970_MOESM5_ESM.docx]

**Additional file 5. Frequency of self-reported menopausal status of female 45 and Up Study participants by single year age at baseline.**

This table illustrates the approach used to determine the age threshold for the conservative approach (here, age ≥57 years at baseline). The frequency is based on female participants who had never used MHT, have not had an oophorectomy nor a hysterectomy, and did not use oral contraceptives at baseline (n=63,742).

| **Menopause status** | **Age at baseline** | | | | | | | | | | | | | | | |
| --- | --- | --- | --- | --- | --- | --- | --- | --- | --- | --- | --- | --- | --- | --- | --- | --- |
|  | **45** | **46** | **47** | **48** | **49** | **50** | **51** | **52** | **53** | **54** | **55** | **56** | **57** | **58** | **59+** | **Total** |
| **Pre-menopause (n)** | 850 | 2032 | 2547 | 2024 | 1550 | 1204 | 838 | 543 | 363 | 197 | 113 | 79 | 52 | 38 | 487 | 12917 |
| ***(% of each age)*** | *83.17* | *74.13* | *68.39* | *59.32* | *48.33* | *36.92* | *27.1* | *18.96* | *13.01* | *7.4* | *3.95* | *3.03* | *2.47* | *1.97* | *1.91* |  |
| **Peri-menopause (n)** | 136 | 542 | 874 | 995 | 1060 | 1185 | 1088 | 901 | 703 | 509 | 343 | 193 | 85 | 37 | 283 | 8934 |
| ***(% of each age)*** | *13.31* | *19.77* | *23.47* | *29.16* | *33.05* | *36.34* | *35.19* | *31.46* | *25.2* | *19.11* | *11.99* | *7.4* | *4.04* | *1.92* | *1.11* |  |
| **Natural menopause (n)** | 36 | 167 | 303 | 393 | 597 | 872 | 1166 | 1420 | 1724 | 1957 | 2404 | 2337 | 1965 | 1850 | 24700 | 41891 |
| ***(% of each age)*** | *3.52* | *6.09* | *8.14* | *11.52* | *18.62* | *26.74* | *37.71* | *49.58* | *61.79* | *73.49* | *84.06* | *89.57* | **93.48** | *96.1* | *96.98* |  |
| **Total** | 1022 | 2741 | 3724 | 3412 | 3207 | 3261 | 3092 | 2864 | 2790 | 2663 | 2860 | 2609 | 2102 | 1925 | 25470 | 63742 |
