# Supplementary material for: Accurate categorisation of menopausal status for research studies: a step-by-step guide and detailed algorithm considering age, self-reported menopause and factors potentially masking the occurrence of menopause
Source: BMC Res Notes. 2022 Mar 4;15:88. doi: 10.1186/s13104-022-05970-z (PMC8895593; doi:10.1186/s13104-022-05970-z)
Supplement: Supplementary file 6 — Additional file 6: Cumulative percentage of female 45 and Up Study participants who had experienced natural menopause by age at baseline. This figure illustrates the approach used to determine the age threshold for the least-conservative approach (here, age ≥ 54 years at baseline). The percentage is based on all female 45 and Up Study participants who had never used MHT, have not had an oophorectomy nor a hysterectomy, and were not using oral contraceptives at baseline (n = 41,891). [file 13104_2022_5970_MOESM6_ESM.docx]

**Additional file 6.** **Cumulative percentage of female 45 and Up Study participants who had experienced natural menopause by age at baseline.**

This figure illustrates the approach used to determine the age threshold for the least-conservative approach (here, age ≥54 years at baseline). The percentage is based on all female 45 and Up Study participants who had never used MHT, have not had an oophorectomy nor a hysterectomy, and were not using oral contraceptives at baseline (n=41,891).
