# Supplementary material for: Accurate categorisation of menopausal status for research studies: a step-by-step guide and detailed algorithm considering age, self-reported menopause and factors potentially masking the occurrence of menopause
Source: BMC Res Notes. 2022 Mar 4;15:88. doi: 10.1186/s13104-022-05970-z (PMC8895593; doi:10.1186/s13104-022-05970-z)

**Additional file 7: Correspondence between directly self-reported, detailed derived and consolidated derived menopausal status in the 45 and Up Study (based on the reference approach, here, age ≥55 years at baseline).**

The diagram illustrates the classification of female 45 and Up Study participants by directly self-reported menopausal status (left vertical bar), detailed derived menopausal status (middle vertical bar) and consolidated derived menopausal status (right vertical bar). The horizontal bands represent the re-classification of participants between the different status assignments, coloured according to the directly self-reported menopausal status. For example, ~19,000 participants had directly self-reported “not sure” status (light blue horizontal bands), of whom ~8,000 were assigned the “no periods due to hysterectomy” detailed derived menopausal status. Of these ~5,500 were then assigned “post-menopause” consolidated derived status in the final step, while ~2,500 were assigned “unknown” consolidated derived status.
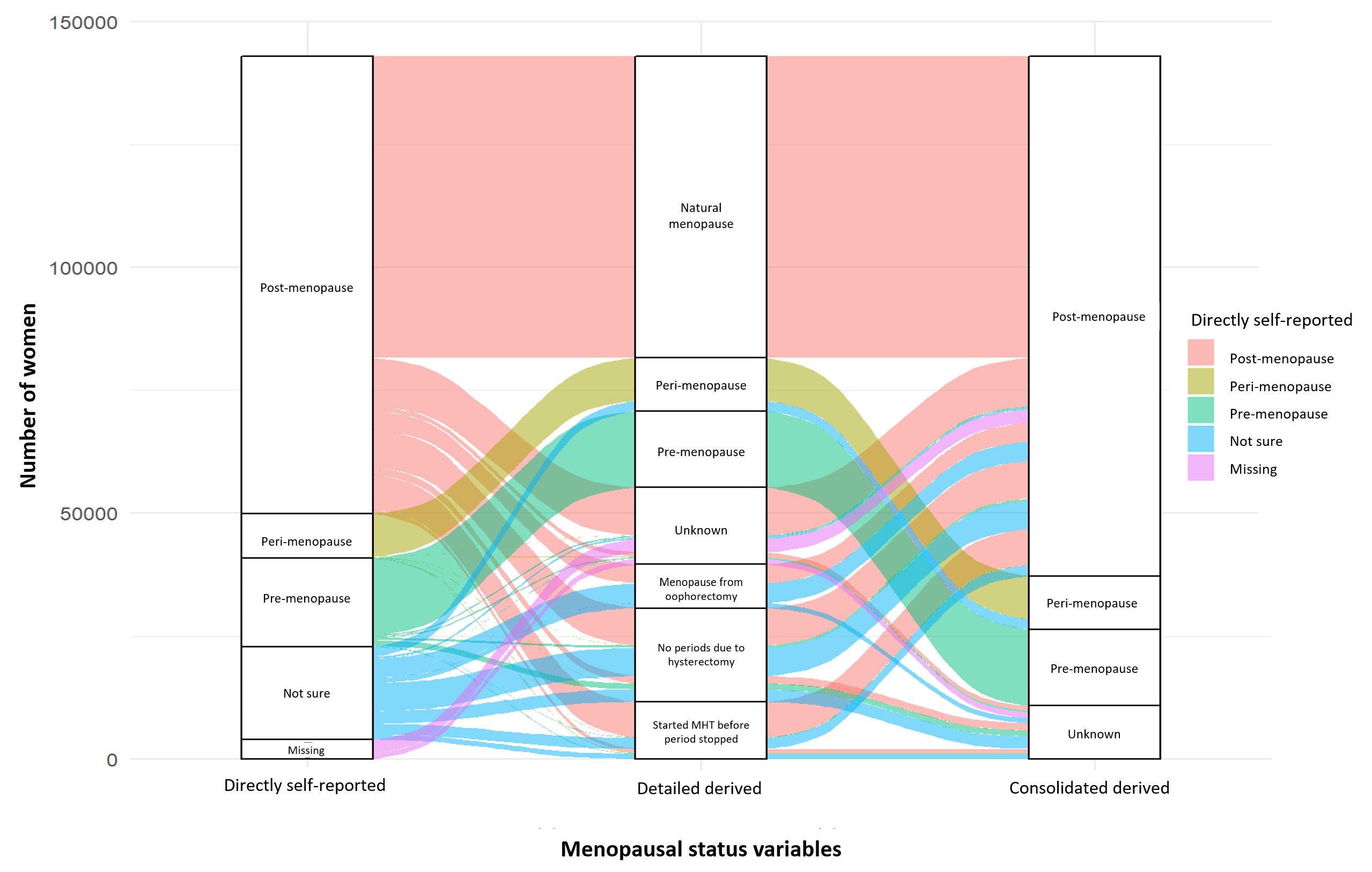

Supplement: Supplementary file 7 — Additional file 7: Correspondence between directly self-reported, detailed derived and consolidated derived menopausal status in the 45 and Up Study (based on the reference approach, here, age ≥ 55 years at baseline). The diagram illustrates the classification of female 45 and Up Study participants by directly self-reported menopausal status (left vertical bar), detailed derived menopausal status (middle vertical bar) and consolidated derived menopausal status (right vertical bar). The horizontal bands represent the re-classification of participants between the different status assignments, coloured according to the directly self-reported menopausal status. For example, ~ 19,000 participants had directly self-reported “not sure” status (light blue horizontal bands), of whom ~ 8,000 were assigned the “no periods due to hysterectomy” detailed derived menopausal status. Of these ~ 5,500 were then assigned “post-menopause” consolidated derived status in the final step, while ~ 2,500 were assigned “unknown” consolidated derived status. [file 13104_2022_5970_MOESM7_ESM.docx]
