# Supplementary material for: Accurate categorisation of menopausal status for research studies: a step-by-step guide and detailed algorithm considering age, self-reported menopause and factors potentially masking the occurrence of menopause
Source: BMC Res Notes. 2022 Mar 4;15:88. doi: 10.1186/s13104-022-05970-z (PMC8895593; doi:10.1186/s13104-022-05970-z)
Supplement: Supplementary file 8 — Additional file 8: Age at menopause, prevalence of hysterectomy, bilateral oophorectomy and current MHT use in the 45 and Up Study compared to other Australian studies. 1 Estimates from InterLACE Study Team. Variations in reproductive events across life: a pooled analysis of data from 505 147 women across 10 countries. Hum Reprod. 2019;34(5):881–93. 2 Estimates from Wilson LF, Pandeya N, Byles J, Mishra GD. Hysterectomy status and all-cause mortality in a 21-year Australian population-based cohort study. Am J Obstet Gynecol. 2019;220(1):83 e1–e11. 3 Estimates from Velentzis LS, Banks E, Sitas F, Salagame U, Tan EH, Canfell K. Use of Menopausal Hormone Therapy and Bioidentical Hormone Therapy in Australian Women 50 to 69 Years of Age: Results from a National, Cross-Sectional Study. PloS one. 2016;11(3):e0146494-e. [file 13104_2022_5970_MOESM8_ESM.docx]

**Additional file 8: Age at menopause, prevalence of hysterectomy, bilateral oophorectomy and current MHT use in the 45 and Up Study compared to other Australian studies.**

| **Study** | **Age of menopause median (IQR)** | **Percentage of women with hysterectomy** | **Percentage of women with hysterectomy that had bilateral oophorectomy** | **Current MHT use** |
| --- | --- | --- | --- | --- |
| 45 and Up Study | 50 (48-53) | 28.5 | 31.0 | 10.0 |
| Australian Longitudinal Study on Women's Health (1946-51 birth cohort) | 51 (49-54) **^1^** | 24.5 **^2^** | 25.6 **^2^** |  |
| Health Aging of Women Study | 50 (48-54) **^1^** |  |  |  |
| Melbourne Collaborative Cohort Study | 50 (48-53) **^1^** |  |  |  |
| The LADY Study |  |  |  | 13.0 **^3^** |

1 Estimates from InterLACE Study Team. Variations in reproductive events across life: a pooled analysis of data from 505 147 women across 10 countries. Hum Reprod. 2019;34(5):881-93

2 Estimates from Wilson LF, Pandeya N, Byles J, Mishra GD. Hysterectomy status and all-cause mortality in a 21-year Australian population-based cohort study. Am J Obstet Gynecol. 2019;220(1):83 e1- e11

3 Estimates from Velentzis LS, Banks E, Sitas F, Salagame U, Tan EH, Canfell K. Use of Menopausal Hormone Therapy and Bioidentical Hormone Therapy in Australian Women 50 to 69 Years of Age: Results from a National, Cross-Sectional Study. PloS one. 2016;11(3):e0146494-e.
